# The Role of Maternal Homocysteine Concentration in Pregnancy Complications: A Systematic Review and Meta-Analysis

**DOI:** 10.3390/jcm15093216

**Published:** 2026-04-23

**Authors:** Ahmed Abu-Zaid, Saeed Baradwan, Majed Saeed Alshahrani, Khalid Khadawardi, Neveen Awadh, Hedaya Albelwi, Heba M. Adly, Saleh A. K. Saleh, Mohammed Abuzaid, Maha Tulbah, Osama Alomar

**Affiliations:** 1 Department of Biochemistry and Molecular Medicine, College of Medicine, Alfaisal University, Riyadh 11533, Saudi Arabia; 2 Department of Obstetrics and Gynecology, King Faisal Specialist Hospital and Research Center, Jeddah 23433, Saudi Arabia; 3 Department of Obstetrics and Gynecology, Faculty of Medicine, Najran University, Najran 66462, Saudi Arabia; 4 Department of Obstetrics and Gynecology, College of Medicine, Umm Al-Qura University, Makkah 21955, Saudi Arabia; 5 Department of Obstetrics and Gynecology, Faculty of Medicine, King Abdulaziz University, Rabigh 25732, Saudi Arabia; 6 Department of Obstetrics and Gynecology, College of Medicine, Tabuk University, Tabuk 47512, Saudi Arabia; 7 Department of Community Medicine and Pilgrims Healthcare, College of Medicine, Umm Al-Qura University, Makkah 21955, Saudi Arabia; 8 Directorate of Institutional Excellence, Batterjee Medical College, Jeddah 21442, Saudi Arabia; 9 Department of Obstetrics and Gynecology, Al Birk General Hospital, Al Birk 63525, Saudi Arabia; 10 Department of Obstetrics and Gynecology, King Faisal Specialist Hospital and Research Center, Riyadh 11211, Saudi Arabia; 11 Department of Obstetrics and Gynecology, College of Medicine, Alfaisal University, Riyadh 11533, Saudi Arabia

**Keywords:** maternal homocysteine, pregnancy complications, placental dysfunction, meta-analysis

## Abstract

**Background:** Adverse pregnancy outcomes such as preeclampsia (PE), preterm birth, low birth weight (LBW), small for gestational age (SGA), and stillbirth are major contributors to maternal and neonatal morbidity and mortality. Elevated maternal homocysteine (Hcy) levels, influenced by genetic, dietary, and lifestyle factors, have been increasingly associated with placental dysfunction and adverse pregnancy outcomes. This review aims to evaluate the link between hyperhomocysteinemia and pregnancy complications to inform clinical practice. **Methods**: A comprehensive search of PubMed, Scopus, Web of Science, and Cochrane Central Library was conducted up to December 2024. Observational studies assessing maternal Hcy levels in relation to pregnancy complications were included. Heterogeneity was measured using the I^2^ statistic, and a random-effects model using the DerSimonian–Laird method was applied to account for study variability. Effect sizes were reported as odds ratios (ORs) with 95% confidence intervals (CIs). **Results**: Thirteen studies were included in this meta-analysis. Elevated maternal Hcy was significantly associated with: PE (OR: 2.49; 95% CI: 1.41–4.40; I^2^ = 96.03%; *n* = 9), preterm birth (OR: 4.01; 95% CI: 1.84–8.72; I^2^ = 91.08%; *n* = 6), fetal loss (OR: 1.76; 95% CI: 1.22–2.52; I^2^ = 41.47%; *n* = 6), SGA (OR: 1.69; 95% CI: 1.35–2.11; I^2^ = 0.00%; *n* = 3), and LBW (OR: 2.46; 95% CI: 1.37–4.43; I^2^ = 77.71%; *n* = 3). **Conclusions**: This review highlights a significant association between elevated maternal Hcy levels and various pregnancy complications. However, given the substantial heterogeneity and reliance on observational evidence, these findings should be interpreted with caution. Future well-designed prospective cohort studies with standardized definitions of hyperhomocysteinemia, consistent timing of exposure assessment across pregnancy trimesters, and adjustment for key confounders are needed to better clarify these associations and underlying mechanisms.

## 1. Introduction

Adverse pregnancy outcomes during pregnancy, such as preeclampsia (PE), preterm birth (PTB), low birth weight (LBW), small for gestational age (SGA), and fetal loss, pose significant health risks for both mothers and their offspring [1,2]. These conditions are major contributors to maternal and neonatal illness and death, with long-term effects on infant development and overall health [3,4].

PE, a pregnancy-related hypertensive disorder, affects 2–8% of pregnancies globally and is a leading cause of complications for both mothers and infants [5,6]. Preterm birth, defined as delivery before 37 weeks of gestation, occurs in approximately 11% of pregnancies and is the primary cause of neonatal mortality [7]. LBW and SGA are associated with increased risks of developmental issues and chronic diseases, affecting survival and quality of life long after infancy [8,9]. Stillbirth, though less common, is a devastating outcome with profound emotional impacts on families [10]. The high prevalence of these complications highlights the critical need for early detection, risk assessment, and targeted interventions to improve outcomes for mothers and newborns [11].

Elevated levels of maternal homocysteine (Hcy), a sulfur-containing amino acid involved in one-carbon metabolism, have been increasingly linked to various pregnancy complications, particularly those related to placental dysfunction [12,13]. Hcy levels are influenced by genetic, dietary, and lifestyle factors [14]. High Hcy concentrations are known to contribute to endothelial dysfunction, oxidative stress, and inflammation [15]. Numerous studies have found associations between hyperhomocysteinemia (elevated Hcy levels) and conditions such as PE, placental abruption, intrauterine growth restriction (IUGR), stillbirth, and miscarriage [16,17,18]. Researchers suggest that high Hcy levels may impair placental vascular development, limiting the transfer of essential nutrients and oxygen to the fetus [19]. This can lead to complications like LBW, SGA infants, and preterm birth [20]. Although gestational diabetes mellitus (GDM) is an important pregnancy outcome, recent systematic reviews [21,22] have already evaluated its association with maternal homocysteine levels. Therefore, to avoid redundancy and maintain a focused scope, GDM was not included in the present review.

While there is substantial evidence in the literature linking hyperhomocysteinemia to pregnancy complications, inconsistencies in study populations, designs, and analytical methods have led to mixed findings [23]. This highlights the need for a comprehensive synthesis of existing data to clarify these relationships and provide a clearer understanding of the underlying mechanisms. This systematic review and meta-analysis aim to summarize current knowledge on the association between elevated maternal Hcy levels and adverse pregnancy outcomes, offering insights to guide future research and clinical practice.

## 2. Methods

### 2.1. Study Protocol and Search Strategy

We have registered our systematic review on the PROSPERO database under the code number CRD420250652459. In accordance with the Preferred Reporting Items for Systematic Reviews and Meta-Analyses (PRISMA) [24] and Meta-analysis of Observational Studies in Epidemiology (MOOSE) [25] guidelines, a systematic review and meta-analysis were undertaken to evaluate possible associations of adverse pregnancy outcomes with high serum Hcy levels. Supplementary File S1 presents the PRISMA checklist, while Supplementary File S2 presents the MOOSE checklist.

We conducted a systematic search of the literature, independently performed by two reviewers, across multiple databases (PubMed, Scopus, Web of Science, and Cochrane Central Library) from their inception until December 2024 (limited to English-language publications). The search strategy included other terms such as the Medical Subject Headings (MeSH) “Homocysteine,” “S-Adenosylhomocysteine,” “Hyperhomocysteinemia,” “Homocystinuria,” and pregnancy outcomes “Premature Birth,” “Stillbirth,” “Intrauterine Fetal Death,” “Low Birth Weight,” and “Fetal Growth Retardation.” To encompass further relevant literature, we identified relevant articles via hand searching reference lists and related articles of PubMed. Details of the search strategy and specific database syntaxes are provided in Supplementary File S3.

### 2.2. Eligibility Criteria and Study Selection

Studies were included in the review if they met the following criteria: (i) they had an observational design, including case–control, cohort, or cross-sectional studies; (ii) they investigated the association between high serum Hcy levels and adverse pregnancy outcomes such as PE, LBW, SGA, PTB, and fetal loss; and (iii) they provided sufficient data on sample size, outcomes, or effect estimates. Studies were excluded if they met any of the following conditions: (i) non-original research articles (e.g., editorials, commentaries, reviews, or conference abstracts without sufficient data); (ii) studies lacking adequate data to extract or calculate effect estimates; (iii) studies with unclear or inappropriate definitions of exposure or outcomes; (iv) duplicate publications or overlapping populations (in which case the most comprehensive or recent study was included); or (v) studies with irretrievable full texts. The study selection process was conducted independently by two reviewers using a standardized approach, with any discrepancies resolved through discussion and consensus.

### 2.3. Data Extraction and Quality Assessment

The data extraction was performed by two authors independently using a predefined form. Discrepancies were resolved by discussion with a third author. The data extracted from the studies included the study title, year of publication, sample size, names of the first authors, characteristics of subjects such as age and BMI, Hcy levels, and study outcomes. For consistency across studies, fetal loss was defined as a composite outcome including miscarriage, IUFD, and stillbirth. However, these outcomes are clinically distinct events occurring at different gestational stages and may involve different underlying mechanisms; therefore, the pooled fetal loss estimate should be interpreted with caution. The first phase of screening involved title and abstract review, while the second phase involved examination of full texts based on inclusion and exclusion criteria that were set prior. In cases of discord, the main author resolved the disagreement and made the final decision. One reviewer assessed the initial selection criteria against all articles, and articles meeting all criteria were included, including other relevant articles identified by searching reference lists from selected studies. Abstracts and unpublished studies were considered during the initial screening; however, only studies with sufficient methodological details and available full texts were included in the final analysis to ensure data reliability. Data extracted were arranged systematically to include information on study design, country of origin, baseline characteristics and Hcy levels (mean ± standard deviation). When necessary, attempts were made to contact corresponding authors of eligible studies to obtain missing or unclear data; however, no additional data were obtained through this process.

The quality of individual studies was assessed using Newcastle-Ottawa scale (NOS), which is a validated tool for evaluating the risk of bias in observational studies [26]. NOS scores assess studies using three domains: the selection of participants, the comparability of groups, and outcome assessment. Each domain has specific criteria and is scored with a maximum score of 9 points. Quality of studies was scored as follows; 7–9 points: low risk of bias (good methodological quality), 4–6 points: moderate risk of bias, and 0–3 points: high risk of bias (low methodological quality). In addition, the overall certainty of the evidence for each outcome was evaluated using the Grading of Recommendations Assessment, Development and Evaluation (GRADE) approach [27]. This approach reduces the certainty of evidence when limitations are identified, including risk of bias, inconsistency, indirectness, imprecision, and possible publication bias. All evaluations were recorded, and any discrepancies were settled by consensus.

### 2.4. Statistical Analysis

Statistical analysis for this meta-analysis was performed using STATA version 17.0 (StataCorp LLC, College Station, TX, USA). The I^2^ test was used to assess heterogeneity across studies, with heterogeneity defined as low (I^2^ = 25%), moderate (I^2^ = 50%), and high (I^2^ = 75%). If there was significant heterogeneity (I^2^ > 50%) [28], a random-effects model using the DerSimonian–Laird method was applied. All data were presented as the pooled odds ratio (OR) and the 95% confidence intervals (CIs), and statistical significance was defined as *p* < 0.05 (two-sided). For the meta-analysis, unadjusted effect estimates were calculated by extracting or deriving 2 × 2 contingency table data (number of exposed and unexposed participants with and without outcomes) from each study. Adjusted estimates were not pooled due to inconsistency in reporting and covariate adjustment across studies. Exposure to high maternal Hcy levels was defined according to the cutoff values reported in each individual study. Due to variability in these thresholds, exposure definitions were harmonized by categorizing participants into high versus reference (normal/low) Hcy groups within each study. Studies reporting continuous Hcy measures were not combined with dichotomous data unless sufficient information was available to derive categorical comparisons. To maintain uniformity, serum Hcy measurements were standardized and expressed in μmol/L (μmol/L). Sensitivity analysis was conducted by leaving out a single study at a time and observing the effect on the summary estimates. Publication bias was assessed using funnel plots and Egger’s regression test; however, these analyses were conducted in an exploratory manner, as the number of included studies for each outcome was fewer than 10, limiting the statistical power of these methods [29].

## 3. Results

### 3.1. Study Selection

**Figure 1:**
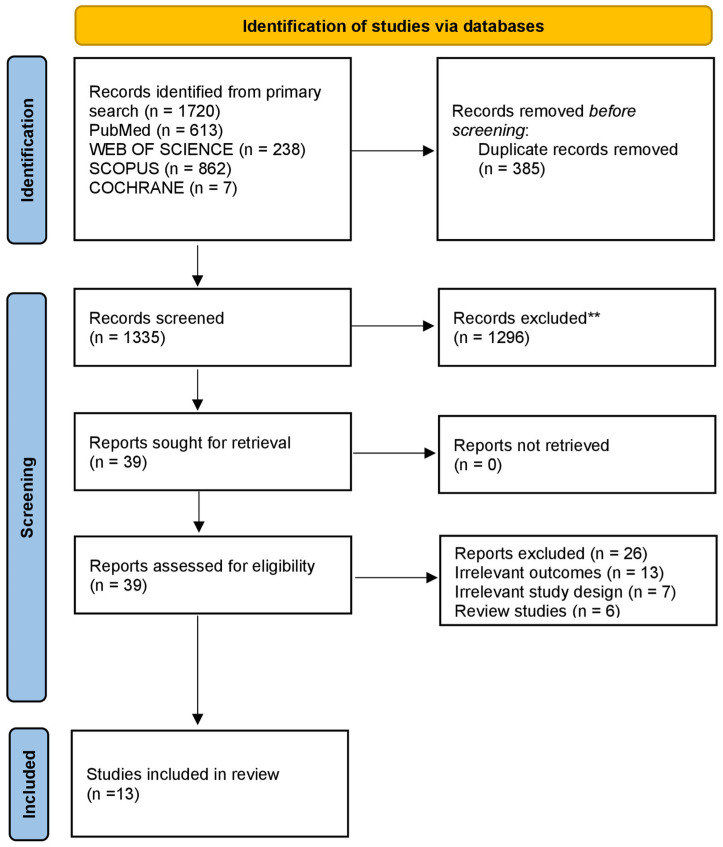
The PRISMA flowchart for literature search and study selection. ** Studies Excluded based on title and abstract screening.

A total of 1720 records were identified through primary database searching. A total of 1355 unique records were first screened for eligibility after the removal of 385 duplicate records. Of these, 1296 records were excluded during the initial screening step for irrelevance. The screening of titles and abstracts, as well as full-text assessment, was conducted independently by two reviewers, with discrepancies resolved through discussion and consultation with a third reviewer when necessary. The remaining 39 reports were requested for retrieval and evaluated for eligibility. After conducting full-text review, 26 reports were excluded for the following reasons: irrelevant outcomes (*n* = 13), inappropriate study design (*n* = 7), and review studies (*n* = 6). A total of 13 studies [12,30,31,32,33,34,35,36,37,38,39,40,41] were included in the systematic review based on inclusion criteria. The study selection process is illustrated in Figure 1.

### 3.2. Main Characteristics of Included Studies

The main characteristics of included studies are presented in Table 1. The 13 observational studies included in the systematic review were carried out in multiple countries (Netherlands, China, Canada, Norway, India, Nigeria, Pakistan, and Indonesia). Sample sizes varied widely between 60 and 14,492 participants. Maternal Hcy concentrations were measured based on different cutoff values, with most studies using a range of ≥8.3 µmol/L to >15 µmol/L, indicating variation in hyperhomocysteinemia definition. Maternal age varied from early to late pregnancy across studies. There was BMI data for majority of the studies, ranging from ~18.5 to 30.4 kg/m^2^. The primary outcomes investigated were adverse pregnancy outcomes including PE, SGA, LBW, preterm birth, fetal growth restriction, and fetal loss. We found significant associations between increased maternal Hcy levels and the risk of these adverse outcomes in a number of studies, suggesting a link of hyperhomocysteinemia to pregnancy complications.

Overall, the findings of the systematic review, as summarized in Table 2, consistently demonstrate that elevated maternal Hcy levels are associated with an increased risk of adverse pregnancy outcomes across diverse study designs and populations. Several cohort studies, including Bergen et al. [30] and Chaudhry et al. [32], reported higher odds of SGA and placenta-mediated complications, while Emil Vollset et al. [40] and Nwogu et al. [36] observed significant associations with PTB and LBW. Similarly, Dodds et al. [12] and Sun et al. [38] found increased risks of PE, particularly severe forms, with elevated Hcy levels. Case–control studies such as Qureshi et al. [37], Mishra et al. [35], and Wiradnyana et al. [41] reported strong associations with PE, PPROM, and PTB, respectively. More recent evidence, including Liu et al. [33] and Thakur et al. [39], further supports a strong relationship between high Hcy and a broad spectrum of placenta-mediated complications. Additionally, Chang et al. [31] highlighted an association between elevated Hcy and miscarriage, extending the impact of hyperhomocysteinemia to early pregnancy loss. Despite some variability in effect sizes and definitions, the overall direction of evidence strongly supports a consistent link between elevated maternal Hcy levels and adverse pregnancy outcomes.

### 3.3. Quality Appraisal of Included Studies

The NOS was used to assess the risk of bias in the included studies, with scores ranging from 5/9 to 8/9. Most studies demonstrated good methodological quality, with eight studies classified as good quality (scoring 7/9 or higher), while five studies were categorized as moderate quality (scoring 5/9 or 6/9). Selection bias was well addressed in most studies, with the majority achieving the highest score (4/4) in this domain. The comparability criterion, which evaluates the adjustment for confounding factors, was adequately met in most studies (scoring 2/2). However, outcome assessment showed some variability, with scores ranging from 1/3 to 2/3, indicating potential limitations in follow-up or outcome measurement in certain studies. Overall, the studies included in this review exhibit a moderate-to-good methodological quality, supporting the robustness of the findings while acknowledging some limitations in outcome assessment.

### 3.4. Meta-Analysis Results

**Figure 2:**
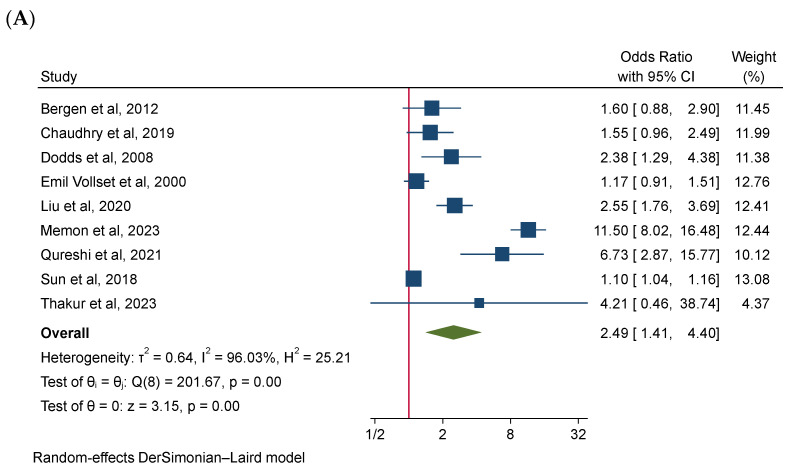

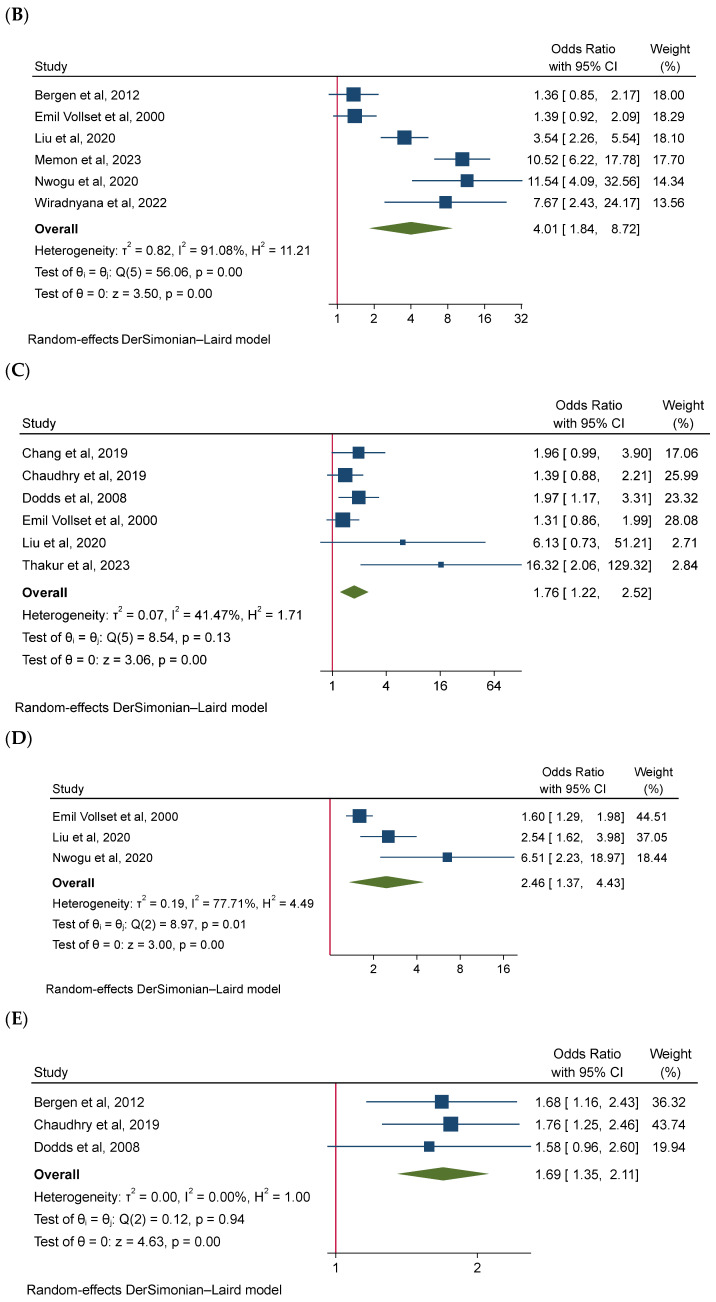
Forest plot showing the association between high maternal homocysteine (Hcy) levels and the risk of preeclampsia (**A**), preterm birth (**B**), fetal loss (**C**), low birth weight (**D**), and small for gestational age (**E**) [12,30,31,32,33,34,35,36,37,38,39,40,41].

The meta-analysis results of our study are presented in Figure 2. In this meta-analysis, we found significant associations between high maternal Hcy levels and negative pregnancy outcomes. Nine studies reported an association between elevated maternal Hcy levels and PE, with a pooled OR of 2.49 (95% CI: 1.41–4.40; *p* = 0.00), indicating high heterogeneity (I^2^ = 96.03%). The pooled OR for the risk of PTB using six studies yielded 4.01 (95% CI: 1.84–8.72; *p* = 0.00, I^2^ = 91.08%). Likewise, six studies examined the relationship between high maternal Hcy levels and fetal loss, demonstrating a combined OR of 1.76 (95% CI: 1.22–2.52; *p* = 0.00) with moderate heterogeneity (I^2^ = 41.47%). In addition, three studies investigated the correlation of elevated maternal Hcy levels and LBW and SGA. The pooled OR for LBW was 2.46 (95% CI: 1.37–4.43; *p* = 0.00, I^2^ = 77.71%) and for SGA, pooled OR of 1.69 (95% CI: 1.35–2.11; *p* = 0.00, I^2^ = 0.00%). While the heterogeneity varied, the pooled meta-analyses of the random-effects models consistently showed significant associations between elevated maternal Hcy levels and elevated risks of LBW and SGA.

### 3.5. Summary of Certainty of Evidence, Sensitivity Analysis, and Publication Bias

The GRADE assessment of our variables is shown in Supplementary File S4. It shows that the strength of evidence for an association between high maternal Hcy levels and PE, preterm birth and LBW is very low as a result of very serious inconsistency or risk of bias. The quality of evidence for fetal loss and SGA is rated as low, indicating uncertainty in the estimates of effect. These limitations notwithstanding, our results also underscore the potential of elevated maternal Hcy levels as risk factors for adverse pregnancy outcomes. We also reported the results of the sensitivity analysis, which examined the effect size after the dropout of each individual study and their impact on the overall effect size for each variable, in Supplementary File S5. For the PE analysis, exclusion of Memon et al. [34] resulted in a reduced effect size (OR: 1.83; 95% CI: 1.30–2.57), but the association remained statistically significant, indicating that the overall findings are robust despite some influence of this study. Moreover, funnel plots for assessing publication bias are provided in Supplementary File S6.

## 4. Discussion

To our best knowledge, this study represents one of the few comprehensive systematic reviews and meta-analyses synthesizing the association between maternal Hcy levels and a broad range of adverse pregnancy outcomes, rather than focusing on a single outcome. Our meta-analysis found a significant association between increased maternal Hcy levels and adverse pregnancy outcomes (including PE, preterm birth, fetal loss, LBW, and SGA). The pooled meta-analysis data revealed that high maternal Hcy levels were significantly associated with increased risks of PE (OR = 2.49), preterm birth (OR = 4.01), fetal loss (OR = 1.76), LBW (OR = 2.46), and SGA (OR = 1.69). These findings highlight the potential role of hyperhomocysteinemia as a marker for pregnancy complications. The heterogeneity seen in some analyses suggests more refinement of these associations is required to define clearer clinical guidelines. While previous reviews have examined specific outcomes or related pathways (e.g., folate and homocysteine metabolism), our study provides an updated and integrated quantitative synthesis across multiple clinically relevant outcomes. However, these associations should be interpreted cautiously because maternal Hcy levels are strongly influenced by folic acid supplementation, serum folate, vitamin B12 status, and other nutritional factors that were not consistently reported or adjusted for across the included studies. Therefore, it remains uncertain whether the observed associations reflect the independent effect of Hcy or broader differences in nutritional status and supplementation practices. Given the substantial heterogeneity and the low to very low certainty of evidence based on GRADE assessment, these findings should be interpreted with caution. Therefore, while maternal Hcy monitoring may be of research interest, its routine use in pregnancy care requires further high-quality evidence.

Our findings are generally consistent with previous studies investigating the relationship between maternal Hcy levels and pregnancy outcomes [17,23,36]. Several observational studies have reported a significant association between hyperhomocysteinemia and PE, LBW, and preterm birth [42,43,44]. Our findings align with those of Bala et al. 2021 [45], who also reported a significant association between elevated maternal Hcy levels and adverse pregnancy outcomes, particularly early pregnancy loss. Similarly, their meta-analysis confirmed that hyperhomocysteinemia is an independent risk factor for pregnancy complications, reinforcing the evidence presented in our study. In addition, our findings align with Zhang et al. 2022 [46], who also reported a significant association between elevated maternal Hcy levels and an increased risk of PE, despite the presence of substantial heterogeneity among studies. Consistent with previous literature, concerns regarding publication bias and low certainty of evidence remain relevant in our analysis. Although funnel plots suggested possible asymmetry and Egger’s test was conducted, these assessments were exploratory due to the limited number of studies (*n* < 10), reducing their statistical power. Moreover, because the pooled estimates were based largely on unadjusted data, residual confounding by folate-related and nutritional variables cannot be excluded. Therefore, the observed associations should be interpreted cautiously, and claims regarding clinical significance should be considered preliminary.

High maternal Hcy levels can have multiple pathophysiological mechanisms that can lead to adverse pregnancy outcomes. One central mechanism is the endothelial dysfunction [15,47] caused by the effect of increased Hcy on the decreased nitric oxide production [48,49], oxidative stress [50], and increased inflammation [51,52], resulting in decreased placental blood flow perfusion and fetal growth impairment [53]. Moreover, Hcy facilitates hypercoagulability by augmenting platelet activation and promoting thrombin formation [54,55], which increases the risk of vascular placental complications like PE and fetal loss [32]. Excessive oxidative damage caused by Hcy may lead to increased trophoblast apoptosis and impaired placentation and thus preterm birth and fetal growth restriction [56]. In addition, an abnormal increase in Hcy impairs the methylation mediation of gene regulation [57,58], which could alter fetal development and increase the risk of LBW and SGA outcomes [44]. All these mechanisms jointly support our observations of significant association between maternal Hcy levels and adverse pregnancy outcomes.

The strengths of our study include the comprehensive and systematic methods used to identify relevant studies, including high-quality observational data across diverse populations. By assessing various adverse pregnancy outcomes while encompassing all major databases, we offer a comprehensive summary of existing evidence. Finally, we assessed the methodological quality of the included studies using the NOS and the GRADE approach to address potential sources of bias. A few limitations must, however, be noted. Firstly, the number of included studies was relatively limited, restricting subgroup analyses that might have clarified sources of heterogeneity. Second, considerable heterogeneity was seen in some of our meta-analyses, and this may be caused by differences in study design, and population characteristics. In addition, variability in homocysteine cutoff values across studies limited our ability to perform subgroup analyses based on exposure thresholds, which may have contributed to residual heterogeneity. Fourth, important nutritional factors such as folate supplementation, serum folate levels, and vitamin B12 status (which are known to influence homocysteine metabolism) were not consistently reported across studies and were not systematically extracted. As a result, we were unable to account for these variables or perform subgroup analyses, and the pooled estimates are based on largely unadjusted data, which may introduce residual confounding. Fifth, the observational nature of included studies limits causal inference, highlighting the requirement for well-conducted prospective cohort studies and randomized controlled trials to validate our findings. Finally, the majority of the included studies originated from Eastern countries, which makes our results less generalizable to Western populations.

## 5. Conclusions

In conclusion, this systematic review and meta-analysis demonstrates an association between elevated maternal Hcy levels and an increased risk of adverse pregnancy outcomes. However, given the observational nature of the included studies, substantial heterogeneity, and the low to very low certainty of evidence, these findings should be interpreted with caution and should not be considered evidence for clinical recommendations. Importantly, because folic acid supplementation, serum folate, vitamin B12 status, and related nutritional factors were not consistently accounted for, it remains uncertain whether Hcy itself is an independent risk factor or a marker of underlying nutritional status. Rather than supporting routine screening or intervention, our results highlight the need for well-designed prospective studies to better clarify the role of maternal Hcy in pregnancy outcomes. Future studies are needed to establish the cut-off values for hyperhomocysteinemia, genetic and dietary factors influencing maternal Hcy levels, and interventional studies to test whether lowering maternal Hcy levels can reduce the risk of pregnancy-related complications. Until such evidence is available, the clinical applicability of maternal Hcy monitoring remains uncertain.

## Figures and Tables

**Table 1:** The Main characteristics of the included studies.

Study	Country	*n*	Hcy Cut-Off µmol/L	Age (Years)	BMI (kg/m^2^)	Main Outcome *	NOS Risk of Bias Assessment
Bergen et al., 2012 [30]	Netherlands	5805	≥8.3	29.8 ± 5.0	23.5 ± 3.4	High Hcy associated with lower placental weight, lower birthweight, and increased risk of SGA	8/9 (Good quality) Selection: 4/4, Comparability: 2/2, Outcome: 2/3
Chang et al., 2019 [31]	China	936	>12.14	27.9 ± 3.3	24.1 ± 4.2	High Hcy associated with reduced ovulation and increased miscarriage	5/9 (Moderate quality) Selection: 2/4, Comparability: 1/2, Outcome: 2/3
Chaudhry et al., 2019 [32]	Canada	7587	>10.0	30.3 ± 5.1	24.9 ± 5.5	High Hcy in early to mid-second trimester associated with increased risk of SGA	8/9 (Good quality) Selection: 4/4, Comparability: 2/2, Outcome: 2/3
Dodds et al., 2008 [12]	Canada	2119	>10.0	NP	NP	High Hcy in early pregnancy associated with increased risk of pregnancy loss and PE, but not with GH or SGA infants.	5/9 (Moderate quality) Selection: 2/4, Comparability: 1/2, Outcome: 2/3
Emil Vollset et al., 2000 [40]	Norway	14,492	>10.7	NP	NP	High Hcy associated with PE, prematurity, very low birth weight, stillbirth, neural tube defects.	5/9 (Moderate quality) Selection: 2/4, Comparability: 1/2, Outcome: 2/3
Liu et al., 2020 [33]	China	1163	>12.60	29.8 ± 5.9	22.2 ± 3.1	Higher Hcy in early pregnancy were associated with PE, preterm birth, and LBW.	8/9 (Good quality)—Selection: 4/4 Comparability: 2/2 Outcome: 2/3
Memon et al., 2023 [34]	India	810	>15	26.5 ± 4.1	22.2 ± 3.1	High Hcy levels in early pregnancy were associated with PE, fetal growth restriction, PTB, placental abruption, and IUFD.	7/9 (Good quality)—Selection: 3/4 Comparability: 2/2 Outcome: 2/3
Mishra et al., 2018 [35]	India	280	>15	24.7 ± 4.5	NP	High Hcy levels and folate deficiency were associated with PPROM.	7/9 (Good quality)—Selection: 3/4 Comparability: 2/2 Outcome: 2/3
Nwogu et al., 2020 [36]	Nigeria	167	>15	28.7 ± 4.3	NP	Elevated maternal Hcy was associated with PTB and LBW in term neonates.	8/9 (Good quality) -Selection: 4/4 Comparability: 2/2 Outcome: 2/3
Qureshi et al., 2021 [37]	Pakistan	132	≥15	26.7 ± 5.8	NP	High Hcy levels significantly associated with PE	5/9 (Moderate quality) Selection: 2/4, Comparability: 1/2, Outcome: 2/3
Sun et al., 2017 [38]	China	9130	NP	31.1 ± 4.2	23.6 ± 3.6	Elevated maternal Hcy in the first trimester is an independent risk factor for severe PE, but not for GH or mild PE.	8/9 (Good quality)—Selection: 4/4 Comparability: 2/2 Outcome: 2/3
Thakur et al., 2023 [39]	India	120	>15	21–25 ^¥^	18.5–24.9 ^¥^	High maternal serum Hcy in the late first trimester are associated with placenta-mediated complications such as abruption, PE, fetal growth restriction, and pregnancy loss.	8/9 (Good quality)—Selection: 4/4 Comparability: 2/2 Outcome: 2/3
Wiradnyana et al., 2022 [41]	Indonesia	60	≥12.85	26.5 (20–40) ^§^	22.5 (20–24) ^§^	High maternal serum Hcy levels increase the risk of PTB by 9.118 times compared to low Hcy levels.	8/9 (Good quality)—Selection: 4/4 Comparability: 2/2 Outcome: 2/3

* BMI: Body Mass Index (kg/m^2^), NOS: Newcastle-Ottawa Scale (used for assessing the quality of non-randomized studies), Hcy: Homocysteine, SGA: Small for Gestational Age, PPROM: Preterm Premature Rupture of Membranes, PE: Preeclampsia, GH: Gestational Hypertension, LBW: Low Birth Weight, NP: Not Provided. ^¥^ Range. ^§^ Interquartile range.

**Table 2:** Key findings of included studies.

Study (Year)	PE	PTB	LBW	SGA	Fetal Loss	Abruption/IUGR/Other
Bergen et al., 2012 [30]	—	—	Reduced BW	OR: 1.70	—	—
Vollset et al., 2000 [40]	OR: 1.32 (0.98–1.77)	OR: 1.38 (1.09–1.75)	OR: 2.01 (1.23–3.27)	—	—	—
Dodds et al., 2008 [12]	RR: 2.7 (1.4–5.0)	—	—	—	RR: 2.1 (1.2–3.6)	—
Chaudhry et al., 2019 [32]	—	—	—	OR: 1.76 (1.25–2.46)	—	Composite: OR 1.63 (1.23–2.16)
Liu et al., 2020 [33]	—	—	—	—	—	APO: aOR 5.89 (4.08–8.51)
Nwogu et al., 2020 [36]	—	Significant assoication	Significant assoication	—	—	—
Memon et al., 2023 [34]	65.18%	28.13%	—	—	IUFD reported	FGR: 34.38%
Mishra et al., 2018 [35]	—	PPROM OR: 8.46	—	—	—	—
Qureshi et al., 2021 [37]	aOR: 4.72	—	—	—	—	—
Thakur et al., 2023 [39]	Severe PE: 21.66%	—	—	—	Pregnancy loss reported	Abruption: 30%; IUGR: 20%
Wiradnyana et al., 2022 [41]	—	aOR: 9.118 (2.581–32.211)	—	—	—	—
Sun et al., 2017 [38]	Severe PE aOR: 1.12 (1.06–1.20)	—	—	—	—	GH null
Chang et al., 2019 [31]	—	—	—	—	Miscarriage ↑	Reproductive outcomes ↑

PE, preeclampsia; PTB, preterm birth; LBW, low birth weight; SGA, small for gestational age; IUFD, intrauterine fetal death; IUGR, intrauterine growth restriction; FGR, fetal growth restriction; GH, gestational hypertension; PPROM, preterm premature rupture of membranes; APO, adverse pregnancy outcomes; OR, odds ratio; aOR, adjusted odds ratio; RR, relative risk; BW, birth weight. ↑ indicates increased risk or higher odds of the outcome associated with elevated maternal homocysteine levels. The percentages shown for some studies (e.g., PE 65.18%, PTB 28.13%, FGR 34.38%, Abruption 30%, IUGR 20%) are event proportions reported within the high-homocysteine group or study population, not pooled meta-analytic effect estimates. They represent the proportion of participants in that individual study who experienced the specified outcome.

## Data Availability

All data are available within the manuscript and its Supplemental Files.

## Supplementary Materials

The following supporting information can be downloaded at: https://www.mdpi.com/article/10.3390/jcm15093216/s1. Supplementary File S1: The PRISMA 2020 checklist. Supplementary File S2: The MOOSE checklist. Supplementary File S3: The literature search strategy. Supplementary File S4: Certainty of evidence of the meta-analyzed outcomes. Supplementary File S5: Sensitivity analysis of high maternal homocysteine (Hcy) levels and the risk of preeclampsia [A], preterm birth [B], fetal loss [C], low birth weight [D], and small for gestational age [E]. Supplementary File S6: Funnel plots of publication bias of high maternal homocysteine (Hcy) levels and the risk of preeclampsia [A], preterm birth [B], fetal loss [C], low birth weight [D], and small for gestational age [E].

## Abbreviations

BMI	Body Mass Index
CI	Confidence Interval
GH	Gestational Hypertension
Hcy	Homocysteine
LBW	Low Birth Weight
MS	Metabolic Syndrome
NOS	Newcastle-Ottawa Scale
OR	Odds Ratio
PE	Preeclampsia
PPROM	Preterm Premature Rupture of Membranes
SGA	Small for Gestational Age
