# Supplementary material for: The Role of Maternal Homocysteine Concentration in Pregnancy Complications: A Systematic Review and Meta-Analysis
Source: J Clin Med. 2026 Apr 23;15(9):3216. doi: 10.3390/jcm15093216 (PMC13163356; doi:10.3390/jcm15093216)
Supplement: Supplementary file 1 [file jcm-15-03216-s001.zip › Supplementary File S2_ 04-03-26.pdf]

Supplementary File S2. The MOOSE checklist.

Item No	Recommendation	Reported on Page No
Reporting of background should include		
1	Problem definition	2
2	Hypothesis statement	3
3	Description of study outcome(s)	3
4	Type of exposure or intervention used	3
5	Type of study designs used	3
6	Study population	3
Reporting of search strategy should include		
7	Qualifications of searchers (eg, librarians and investigators)	3
8	Search strategy, including time period included in the synthesis and key words	3
9	Effort to include all available studies, including contact with authors	3
10	Databases and registries searched	3
11	Search software used, name and version, including special features used (eg, explosion)	3
12	Use of hand searching (eg, reference lists of obtained articles)	3
13	List of citations located and those excluded, including justification	3
14	Method of addressing articles published in languages other than English	4
15	Method of handling abstracts and unpublished studies	4
16	Description of any contact with authors	4
Reporting of methods should include		
17	Description of relevance or appropriateness of studies assembled for assessing the hypothesis to be tested	4
18	Rationale for the selection and coding of data (eg, sound clinical principles or convenience)	4
19	Documentation of how data were classified and coded (eg, multiple raters, blinding and interrater reliability)	4
20	Assessment of confounding (eg, comparability of cases and controls in studies where appropriate)	4
21	Assessment of study quality, including blinding of quality assessors, stratification or regression on possible predictors of study results	4
22	Assessment of heterogeneity	4
23	Description of statistical methods (eg, complete description of fixed or random effects models, justification of whether the chosen models account for predictors of study results, dose-response models, or cumulative meta-analysis) in sufficient detail to be replicated	4
24	Provision of appropriate tables and graphics	4
Reporting of results should include		
25	Graphic summarizing individual study estimates and overall estimate	8
26	Table giving descriptive information for each study included	6
27	Results of sensitivity testing (eg, subgroup analysis)	6
28	Indication of statistical uncertainty of findings	9

Item No	Recommendation	Reported on Page No
Reporting of discussion should include		
29	Quantitative assessment of bias (eg, publication bias)	N/A
30	Justification for exclusion (eg, exclusion of non-English language citations)	3
31	Assessment of quality of included studies	7
Reporting of conclusions should include		
32	Consideration of alternative explanations for observed results	10
33	Generalization of the conclusions (ie, appropriate for the data presented and within the domain of the literature review)	10
34	Guidelines for future research	11
35	Disclosure of funding source	11
