# Supplementary material for: The Role of Maternal Homocysteine Concentration in Pregnancy Complications: A Systematic Review and Meta-Analysis
Source: J Clin Med. 2026 Apr 23;15(9):3216. doi: 10.3390/jcm15093216 (PMC13163356; doi:10.3390/jcm15093216)
Supplement: Supplementary file 1 [file jcm-15-03216-s001.zip › Supplementary File S3_04-03-26.pdf]

Supplementary File S3. The literature search strategy.

Maternal serum homocysteine levels correlation with infant and pregnancy outcomes: A systematic review and dose–response meta-analysis

Groups	Descriptors
Outcome	“Pregnancy Outcome” OR “Premature Birth” OR “Stillbirth” OR “Intrauterine Fetal Death” OR “IUFD” OR “Infant, Low Birth Weight” OR “Low Birth Weight” OR “LBW” OR “Fetal Growth Retardation” OR “IUGR” OR “Fetal Macrosomia” OR “Infant, Small for Gestational Age” OR “SGA” OR “Asphyxia Neonatorum” OR “Birth Asphyxia”
Exposure	“Homocysteine” OR “S-Adenosylhomocysteine” OR “Hyperhomocysteinemia” OR “Homocystinuria” OR “Homocystin*”

PUBMED

Number of localized studies: 613
Limits: -
Number of studies after applying limits: 613

	Descriptors	Number of studies reached
**#1**	"pregnancy outcome"[MeSH Terms] OR ("pregnancy"[All Fields] AND "outcome"[All Fields]) OR "pregnancy outcome"[All Fields] OR ("premature birth"[MeSH Terms] OR ("premature"[All Fields] AND "birth"[All Fields]) OR "premature birth"[All Fields]) OR ("stillbirth"[MeSH Terms] OR "stillbirth"[All Fields] OR "stillbirths"[All Fields]) OR ("stillbirth"[MeSH Terms] OR "stillbirth"[All Fields] OR ("intrauterine"[All Fields] AND "fetal"[All Fields] AND "death"[All Fields]) OR "intrauterine fetal death"[All Fields] OR "fetal death"[MeSH Terms] OR ("fetal"[All Fields] AND "death"[All Fields]) OR "fetal death"[All Fields]) OR "IUFD"[All Fields] OR ("infant, low birth weight"[MeSH Terms] OR ("infant"[All Fields] AND "low"[All Fields] AND "birth"[All Fields] AND "weight"[All Fields]) OR "low birth weight infant"[All Fields] OR "infant low birth weight"[All Fields]) OR ("infant, low birth weight"[MeSH Terms] OR ("infant"[All Fields] AND "low"[All Fields] AND "birth"[All Fields] AND "weight"[All Fields]) OR "low birth weight infant"[All Fields] OR ("low"[All Fields] AND "birth"[All Fields] AND "weight"[All Fields]) OR "low birth weight"[All Fields]) OR "LBW"[All Fields] OR ("foetal growth retardation"[All Fields] OR "fetal growth retardation"[MeSH Terms] OR ("fetal"[All Fields] AND "growth"[All Fields] AND "retardation"[All Fields]) OR "fetal growth retardation"[All Fields]) OR ("fetal growth retardation"[MeSH Terms] OR ("fetal"[All Fields] AND "growth"[All Fields] AND "retardation"[All Fields]) OR "fetal growth retardation"[All Fields] OR "iugr"[All Fields]) OR ("foetal macrosomia"[All Fields] OR "fetal macrosomia"[MeSH Terms] OR ("fetal"[All Fields] AND "macrosomia"[All Fields]) OR "fetal macrosomia"[All Fields]) OR ("infant, small for gestational age"[MeSH Terms] OR ("infant"[All Fields] AND "small"[All Fields] AND "gestational"[All Fields] AND "age"[All Fields]) OR "small for gestational age infant"[All Fields] OR "infant small for gestational age"[All Fields]) OR "SGA"[All Fields] OR ("asphyxia neonatorum"[MeSH Terms] OR ("asphyxia"[All Fields] AND "neonatorum"[All Fields]) OR "asphyxia neonatorum"[All Fields]) OR	332606

	("asphyxia neonatorum"[MeSH Terms] OR ("asphyxia"[All Fields] AND "neonatorum"[All Fields]) OR "asphyxia neonatorum"[All Fields] OR ("birth"[All Fields] AND "asphyxia"[All Fields]) OR "birth asphyxia"[All Fields])	
**#2**	"homocystein"[All Fields] OR "homocysteine"[MeSH Terms] OR "homocysteine"[All Fields] OR "homocysteine s"[All Fields] OR "homocysteines"[All Fields] OR "s adenosylhomocysteine"[MeSH Terms] OR "s adenosylhomocysteine"[All Fields] OR "s adenosylhomocysteine"[All Fields] OR "hyperhomocysteinemia"[MeSH Terms] OR "hyperhomocysteinemia"[All Fields] OR "hyperhomocysteinemias"[All Fields] OR "homocystinuria"[MeSH Terms] OR "homocystinuria"[All Fields] OR "homocystinurias"[All Fields] OR "homocystin*"[All Fields]	34184
**#3**	**#1** AND **#2**	613

WEB OF SCIENCE

Number of localized studies: 270
Limits: documents types (articles)
Number of studies after applying limits: 238

	**Descriptors**	Number of studies reached
**#1**	TS=(“ Pregnancy Outcome”) OR TS=(“ Premature Birth”) OR TS=(“ Stillbirth”) OR TS=(“ Intrauterine Fetal Death”) OR TS=(“ IUFD”) OR TS=(“ Infant, Low Birth Weight”) AND TS=(“ Low Birth Weight”) OR TS=(“ LBW”) OR TS=(“ Fetal Growth Retardation”) OR TS=(“ IUGR”) OR TS=(“ Fetal Macrosomia”) AND TS=(“ Infant, Small for Gestational Age”) OR TS=(“ SGA”) OR TS=(“ Asphyxia Neonatorum”) OR TS=(“ Birth Asphyxia”)	66412
**#2**	TS=(“Homocysteine”) OR TS=(“S-Adenosylhomocysteine”) OR TS=(“Hyperhomocysteinemia”) OR TS=(“Homocystinuria”) OR TS=(“Homocystin”)*	46260
**#3**	**#1** AND **#2**	270

SCOPUS

Number of localized studies: 1211
Limits: *document type* (article and article in press)
Number of studies after applying limits: 862

	**Descriptors**	Number of studies reached
**#1**	(TITLE-ABS-KEY (birth AND asphyxia)) OR (TITLE-ABS-KEY (asphyxia AND neonatorum)) OR (TITLE-ABS-KEY (sga)) OR (TITLE-ABS-KEY (infant, AND small AND for AND gestational AND age)) OR (TITLE-ABS-KEY (fetal AND macrosomia)) OR (TITLE-ABS-KEY (pregnancy AND outcome)) OR (TITLE-ABS-KEY (premature AND birth)) OR (TITLE-ABS-KEY (stillbirth)) OR (TITLE-ABS-KEY (intrauterine AND fetal AND death)) OR (TITLE-ABS-KEY (iufd)) OR (TITLE-ABS-KEY (infant, AND low AND birth AND weight)) OR (TITLE-ABS-KEY (low AND birth AND weight)) OR (TITLE-ABS-KEY (lbw)) OR (TITLE-ABS-KEY (fetal AND growth AND retardation)) OR (TITLE-ABS-KEY (iugr))	436157
**#2**	(TITLE-ABS-KEY (homocysteine)) OR (TITLE-ABS-KEY (s-adenosylhomocysteine)) OR (TITLE-ABS-KEY (hyperhomocysteinemia)) OR (TITLE-ABS-KEY (homocystinuria)) OR (TITLE-ABS-KEY (homocystin*))	50743
**#3**	**#1** AND **#2**	1211

COCHRANE

Number of localized studies: 7
Limits: -
Number of studies after applying limits: 7

	**Descriptors**	Number of studies reached
**#1**	Me ("Pregnancy Outcome"):ti,ab,kw or ("Premature Birth"):ti,ab,kw or ("Stillbirth"):ti,ab,kw or ("Intrauterine Fetal Death"):ti,ab,kw or ("IUFD"):ti,ab,kw or ("Infant, Low Birth Weight"):ti,ab,kw or ("Low Birth Weight"):ti,ab,kw or ("LBW"):ti,ab,kw or ("Fetal Growth Retardation"):ti,ab,kw or ("IUGR"):ti,ab,kw or ("Fetal Macrosomia"):ti,ab,kw or ("Infant, Small for Gestational Age"):ti,ab,kw or ("SGA"):ti,ab,kw or ("Asphyxia Neonatorum"):ti,ab,kw or ("Birth Asphyxia"):ti,ab,kw	13241
**#2**	Me ("Uric Acid") or ("Urate"):ti,ab,kw or ("Trioxopurine"):ti,ab,kw or ("Hyperuricemia"):ti,ab,kw or ("Hyperuri*"):ti,ab,kw or ("Hypouri*"):ti,ab,kw	740
**#3**	**#1** AND **#2**	7
