# Supplementary material for: The Role of Maternal Homocysteine Concentration in Pregnancy Complications: A Systematic Review and Meta-Analysis
Source: J Clin Med. 2026 Apr 23;15(9):3216. doi: 10.3390/jcm15093216 (PMC13163356; doi:10.3390/jcm15093216)
Supplement: Supplementary file 1 [file jcm-15-03216-s001.zip › Supplementary File S4_04-03-26.pdf]

Supplementary File S4. Certainty of evidence of the meta-analyzed outcomes.

Certainty assessment							Effect	Certainty	Importance
№ of studies	Study design	Risk of bias	Inconsistency	Indirectness	Imprecision	Other considerations	Relative (95% CI)		
**Preeclampsia**									
9	non-randomised studies	not serious	serious[a]	not serious	not serious	none	**OR 2.49** (1.41 to 4.40)	⨁◯◯◯ Very low	Important
**Preterm birth**									
6	non-randomised studies	not serious	serious[a]	not serious	not serious	none	**OR 4.01** (1.84 to 8.72)	⨁◯◯◯ Very low	Important
**Fetal loss**									
6	non-randomised studies	not serious	not serious	not serious	not serious	none	**OR 1.96** (1.17 to 3.31)	⨁⨁◯◯ Low	Important
**Low Birth Weight**									
3	non-randomised studies	not serious	not serious	not serious	not serious	none	**OR 2.54** (1.62 to 3.98)	⨁⨁◯◯ Low	Important
**Small for Gestational Age**									
3	non-randomised studies	not serious	not serious	not serious	not serious	none	**OR 1.69** (1.35 to 2.11)	⨁⨁◯◯ Low	Important

CI: confidence interval; **OR:** odds ratio

Explanations

a. Serious inconsistency since I^2> 60%. Downgraded.
