# Supplementary material for: The Role of Maternal Homocysteine Concentration in Pregnancy Complications: A Systematic Review and Meta-Analysis
Source: J Clin Med. 2026 Apr 23;15(9):3216. doi: 10.3390/jcm15093216 (PMC13163356; doi:10.3390/jcm15093216)
Supplement: Supplementary file 1 [file jcm-15-03216-s001.zip › Supplementary File S5_04-03-26.pdf]

Supplementary File S5. Sensitivity analysis of high maternal homocysteine (Hcy) levels and the risk of preeclampsia **[A]**, preterm birth **[B]**, fetal loss **[C]**, low birth weight **[D]**, and small for gestational age **[E]**.

[A]

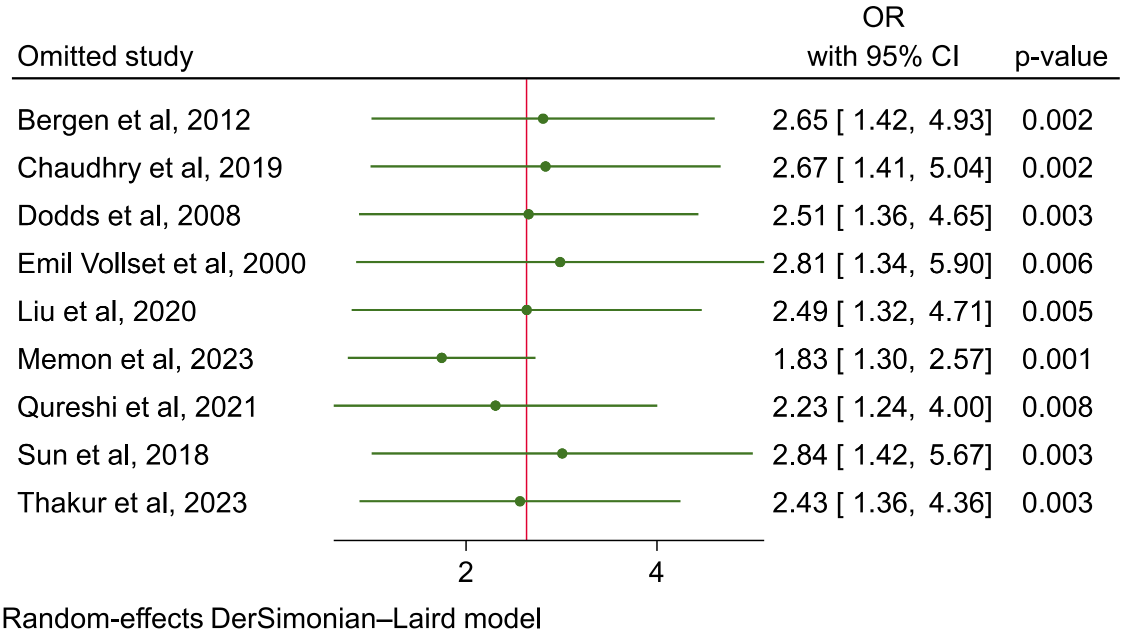

[B]

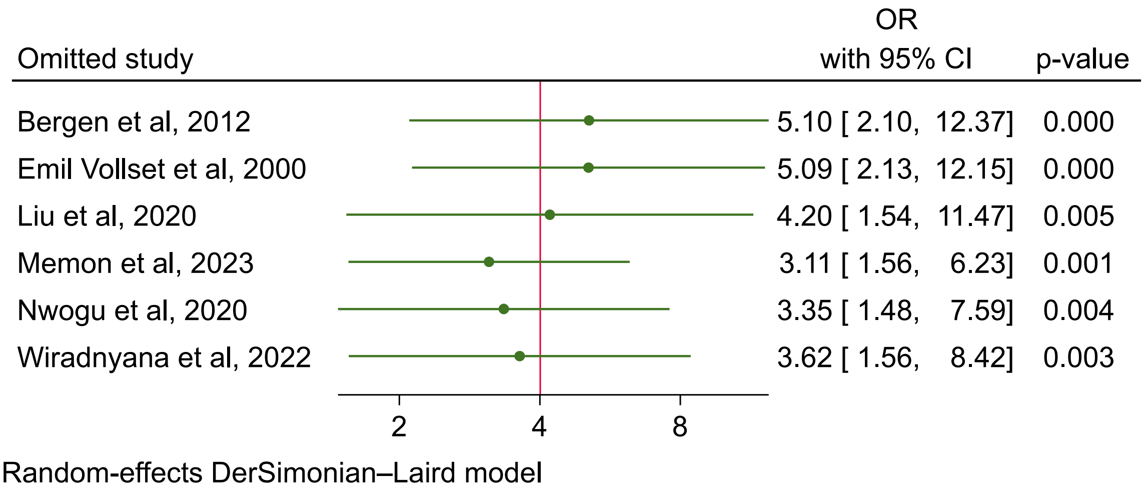

[C]

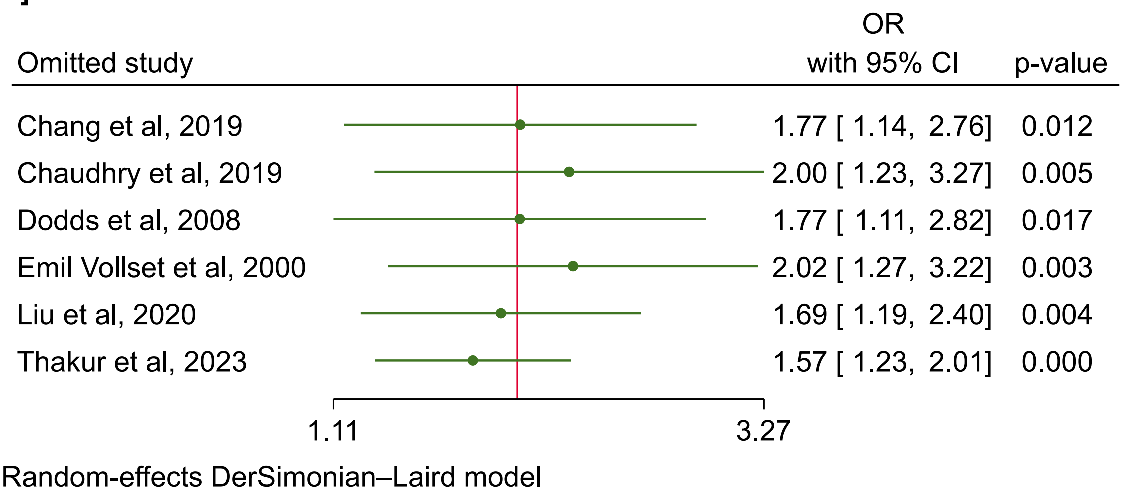

[D]

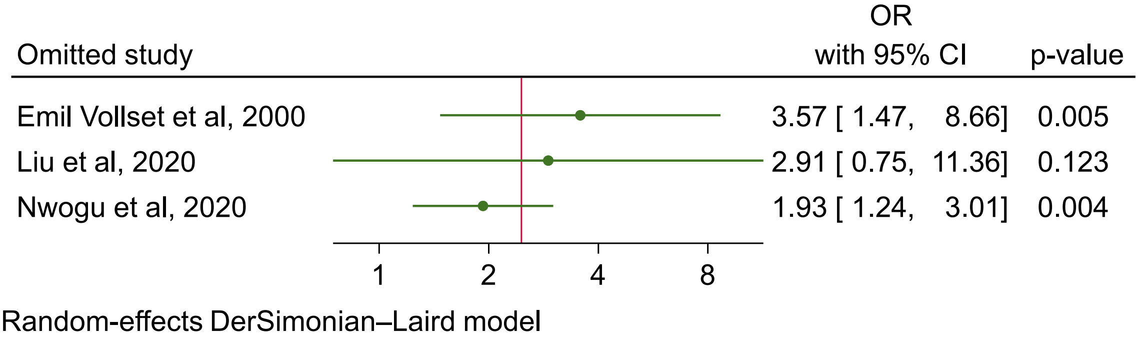
Omitted study
OR with 95% CI
p-value
Emil Vollset et al, 2000
3.57 [1.47, 8.66]
0.005
Liu et al, 2020
2.91 [0.75, 11.36]
0.123
Nwogu et al, 2020
1.93 [1.24, 3.01]
0.004
1
2
4
8
Random-effects DerSimonian–Laird model

[E]

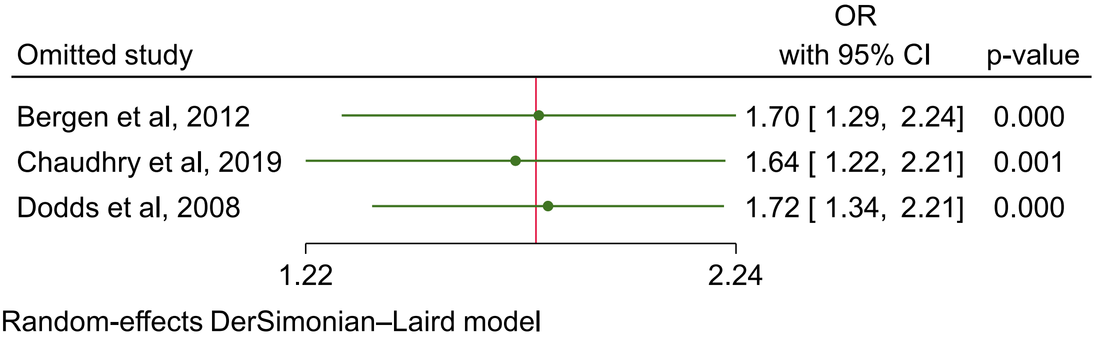
Omitted study
OR with 95% CI
p-value
Bergen et al, 2012
1.70 [1.29, 2.24]
0.000
Chaudhry et al, 2019
1.64 [1.22, 2.21]
0.001
Dodds et al, 2008
1.72 [1.34, 2.21]
0.000
1.22
2.24
Random-effects DerSimonian–Laird model
