# Supplementary material for: The Role of Maternal Homocysteine Concentration in Pregnancy Complications: A Systematic Review and Meta-Analysis
Source: J Clin Med. 2026 Apr 23;15(9):3216. doi: 10.3390/jcm15093216 (PMC13163356; doi:10.3390/jcm15093216)
Supplement: Supplementary file 1 [file jcm-15-03216-s001.zip › Supplementary File S6_04-03-26.pdf]

Supplementary File S6. Funnel plots of publication bias of high maternal homocysteine (Hcy) levels and the risk of preeclampsia **[A]**, preterm birth **[B]**, fetal loss **[C]**, low birth weight **[D]**, and small for gestational age **[E]**.

[A]

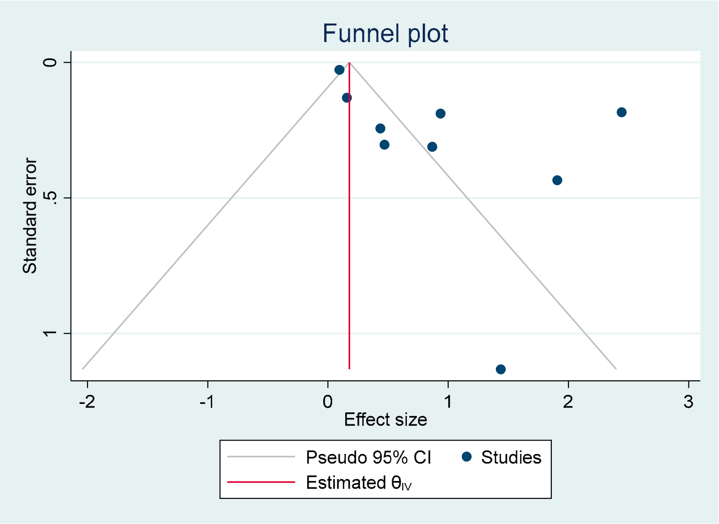

[B]

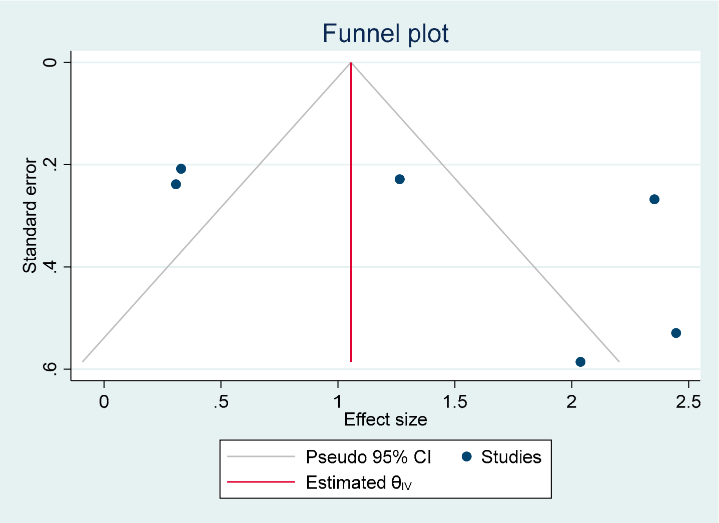

[C]

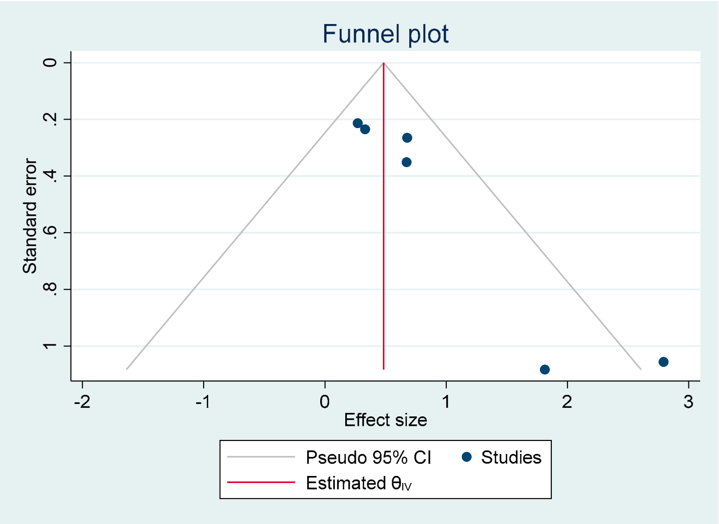

[D]

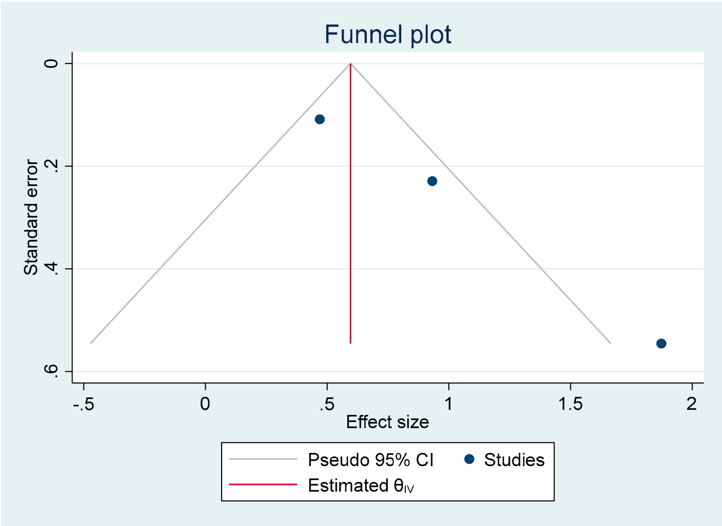
Funnel plot
Standard error
0
.2
.4
.6
-.5
0
.5
1
1.5
2
Effect size
Pseudo 95% CI
Studies
Estimated θIV

[E]

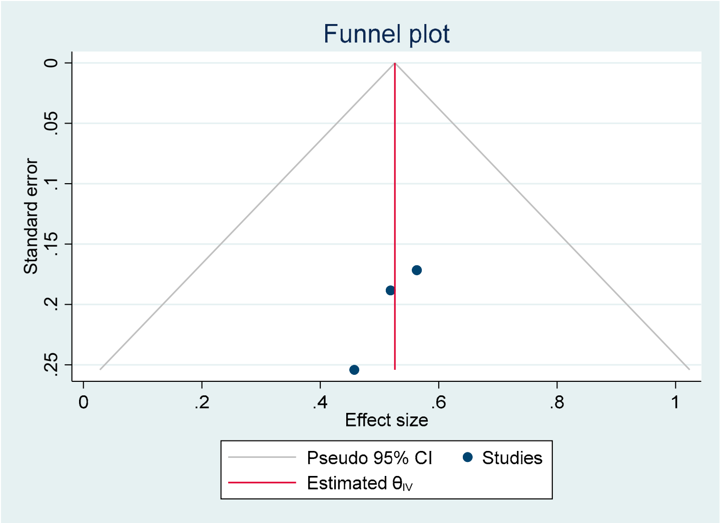
Funnel plot
Standard error
0
.05
.1
.15
.2
.25
0
.2
.4
.6
.8
1
Effect size
Pseudo 95% CI
Studies
Estimated θIV
